# The Association Between Neck Pain and Psychological Distress Experienced by King Abdulaziz University Students: A Cross-Sectional Study

**DOI:** 10.7759/cureus.35685

**Published:** 2023-03-02

**Authors:** Mohammed S Alghamdi, Abdullah F Alghamdi, Asim M Almalawi, Raed A Alsulami, Hassan A Hazazi, Abdulrahman A Al Ghashmari, Ali S Al Dawais, Emad Salawati

**Affiliations:** 1 Family Medicine, King Abdulaziz University Hospital, Jeddah, SAU

**Keywords:** psychological distress, bmi, neck pain, anxiety, depression

## Abstract

Background

Musculoskeletal discomfort resulting from soft tissue injuries to muscles, bones, nerves, tendons, joints, or cartilage is referred to as musculoskeletal disorders. Neck pain is a common musculoskeletal condition with a significant socioeconomic impact on patients. Previous literature has linked the onset of neck pain to various factors, including psychological factors that may affect musculoskeletal disorders (MSDs), similarly to physical factors. Psychological conditions, including anxiety and depression, may result in MSDs. Limited studies on the relationship between neck pain and psychological distress have been conducted among undergraduate students in Jeddah. The study aimed to investigate the relationship between neck pain and psychological distress. Additionally, the study examined the risk factors for developing neck pain, depression, and anxiety in King Abdulaziz University (KAU) undergraduate students.

Method

This cross-sectional study was conducted in November 2022 at KAU in Jeddah, Saudi Arabia, by distributing a google forms survey among undergraduate university students in KAU, excluding graduate students and students who did not agree to participate. We received 509 responses; each respondent gave written consent and participated in the study.

Result

Neck pain prevalence was 50.7% of all students (95% CI, 46.3-55.1). Significantly higher neck pain scores were observed in women (p<0.001), in students who did little or no exercise, and in those who drank coffee more frequently >3 cups daily. Anxiety (p<0.001) and depression (p<0.001) scores were also positively and significantly correlated with neck pain scores. The results of the association analysis revealed that women had significant scores for anxiety (p<0.001) and depression (p<0.001). Female sex (p<0.001) and increased neck pain score (p<0.001) were independent risk factors for anxiety. Higher neck pain scores were also associated with depression (p<0.001).

Conclusion

Our study showed that anxiety and depression significantly impact neck pain. Furthermore, the increased score of depression and anxiety indicates worsening neck pain.

## Introduction

Muscular or skeletal discomfort that results from soft tissue injuries to muscles, bones, nerves, tendons, joints, or cartilage is referred to as musculoskeletal disorder (MSD) [1]. MSDs affect many people from various aspects of society, especially those whose jobs demand a lot of physical and mental effort [2]. The Global Burden of Disease (GBD) study from 2017 ranked musculoskeletal disorders as the second-largest cause of disability worldwide [2]. One of the most prevalent musculoskeletal conditions is neck pain, a significant social and economic burden on affected people, their families, and communities. Additionally, it is becoming an important health concern [3,4].

The previous literature reviews demonstrate that the onset of neck pain has been associated with many factors, including socio-demographic factors like gender, age, body mass index (BMI), and social and behavioral factors such as smoking, drinking coffee, physical activity, stress, and sleeping hours [5,6].

Recent studies have suggested that psychological risk factors may affect MSDs similarly to physical risk factors. It has been demonstrated that psychological conditions, including anxiety, high level of distress, and depression, can result in MSDs [7,8]. Psychological factors such as anxiety and depression can be assessed as complementary therapeutic strategies for neck pain and physical disability. These factors play a significant role in the onset of pain-related disability and impact how pain is perceived [9,10].

A study conducted among university students and the general population found a significant relationship suggesting that depression and anxiety were significantly linked with increasing neck pain [11]. Another study in China revealed an association between musculoskeletal symptoms and psychological distress, especially in the neck region [12]. A survey done locally in Saudi Arabia showed that 68.4% of the patients with neck pain suffer from anxiety, while 55.7% have depression [13].

Limited studies have been conducted on the association between neck pain and psychological distress among undergraduate students in Jeddah. Hence, the purpose of this study is to assess the correlation between neck pain and psychological distress and investigate the associated risk factors for developing neck pain, depression, and anxiety in undergraduate students of King Abdulaziz University (KAU).

## Materials and methods

Aim and objective

This study determines the link between neck pain and psychological distress. Additionally, the study investigates the amount of neck pain impairment and psychological distress among undergraduate students at King Abdulaziz University in Jeddah, Saudi Arabia, as well as the prevalence and risk variables connected with the condition. 

Study design and setting

This cross-sectional study was conducted in November 2022 at King Abdulaziz University in Jeddah, Saudi Arabia, using a google forms survey endorsed by the KAUH Research Ethics Committee. The referral number is (497-22), with (HA-02-J-008) being the National Committee of Bio & Med registration number. All participants gave their informed consent.

Study population and sample size

Our population consisted of all undergraduate students at King Abdulaziz University in November 2022, excluding graduate students and students who did not agree to participate. The total population size was 117,096. According to King Abdulaziz University student statistics, the minimum percentage of sample size needed for this study was calculated as 0.33% with a 95% confidence level and a 5% margin of error [14]. That has been calculated using the Raosoft® sampling calculator. We received 509 responses. Each respondent gave written consent and participated in the study.

Data collection and definition of variables

Using Google Forms, the survey was emailed to King Abdulaziz University students. The survey included demographics (sex, age, height, weight, and marital status) and behaviors (smoking, exercise, and coffee consumption). Additionally, assessments of neck disability using the Neck Pain Disability Index Questionnaire (NDI) and anxiety and depression using the Hospital Anxiety and Depression Scale (HADS) were done. The current study defined neck pain as self-reported pain ranging from mild to severe [15,16]. We used a validated Arabic version of the NDI disability scale [17], which consists of 10 items that assess physical function restrictions (i.e., lifting, driving) as well as impairments in bodily function (e.g., reading and concentration). Six possible answers to each question range from zero (no disability) to five (severe disability.) The maximum raw score is 50. The various categories of disability levels include; Minimal Disability 0-10, Moderate disability 11-20, Severe Disability 21-30, Crippled 31-40, and 41-50 Bed-bound or exaggerated symptoms. A self-screening tool called HADS is used to identify and categorize the degree of anxiety and depression. We used the Terkawi et al. verified HADS in Arabic [18] comprising 14 questions and 2 subscales for anxiety (HAD-A) and depression (HAD-D), each having seven questions. The maximum scores for both anxiety and depression are 21. Each question is worth four points, and the total points from 0-7 are considered normal, 8-10 borderline and 11-21 indicate anxiety or depression.

Data entry and data analysis

Data entry was done with Microsoft Excel 2020, and statistical analysis was carried out using RStudio (R version 4.1.1). Normality testing was carried out for numerical variables, revealing non-normally distributed data for all the variables (p < 0.001). Numerical variables were expressed as a median and interquartile range, whereas categorical data were presented as frequencies and percentages. A Wilcoxon rank sum test or Kruskal-Wallis rank sum test was used to assess the differences in scores of neck pain, anxiety, and depression across different socio-demographic groups. Bivariate correlations between numerical scores were explored using Spearman’s correlation analysis, and the outcomes were depicted in scatterplots. The significantly associated variables with anxiety and depression were further entered into the multivariate linear regression model (using each outcome in a separate model). The results were expressed as beta coefficients and their respective 95% confidence intervals (95% CIs). No other selection criteria were applied to include the independent variables in the multiple regression model. A p-value of <0.05 indicates statistical significance.

## Results

Sociodemographic characteristics

The data of 509 students were analyzed in the current study. Men represented 56.0% of the sample; most were single (97.4%). The median (IQR) age of students was 22.0 years (20.0, 23.0), and the median (IQR) BMI was 23.2 Kg/m2 (20.1, 28.0). One-quarter of students were not performing exercise at all (25.0%), and 12.0% were smokers. The remaining characteristics are demonstrated in Table 1.

**Table 1:** Sociodemographic characteristics *the variables are based on 61 smokers with 4 missing values.

Parameter	Category	Results
Sex	Men	285 (56.0%)
	Women	224 (44.0%)
Age	<20 years	81 (15.9%)
	20 to 23 years	337 (66.2%)
	>23 years	91 (17.9%)
Height (cm)	Median (IQR)	168.0 (159.0, 173.5)
Weight (Kg)	Median (IQR)	65.0 (54.0, 80.0)
BMI (Kg/m^2^)	Median (IQR)	23.2 (20.1, 28.0)
BMI categories	Underweight	77 (15.1%)
	Healthy weight	243 (47.7%)
	Overweight	101 (19.8%)
	Obese	88 (17.3%)
Marital status	Single	496 (97.4%)
	Married	13 (2.6%)
Smoker	Yes	61 (12.0%)
Number of cigarettes per day*	Median (IQR)	10.0 (6.0, 20.0)
Years of smoking*	Median (IQR)	5.0 (4.0, 7.0)
Do you exercise?	Yes, regularly	92 (18.1%),
	Yes, but occasionally	290 (57.0%)
	No, not at all	127 (25.0%)
Number of cups of coffee per week	None	111 (21.8%)
	1 to 3	138 (27.1%)
	4 to 7	163 (32.0%)
	>7	97 (19.1%)

Descriptive and reliability analyses of the used scales

**Figure 1:**
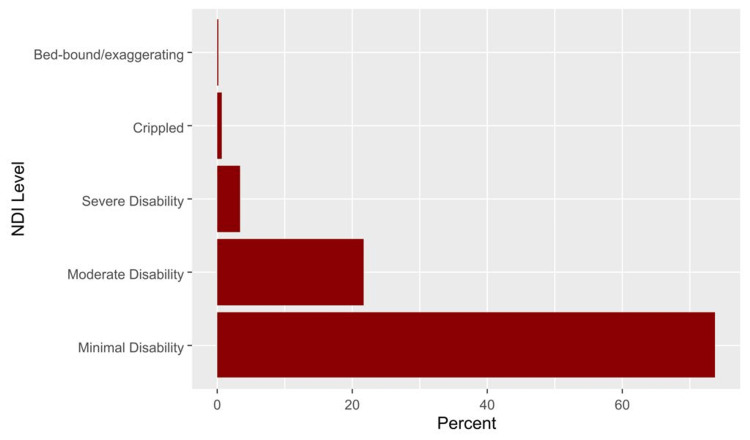
The percentages of different levels of neck pain disability based on the neck disability index.

**Figure 2:**
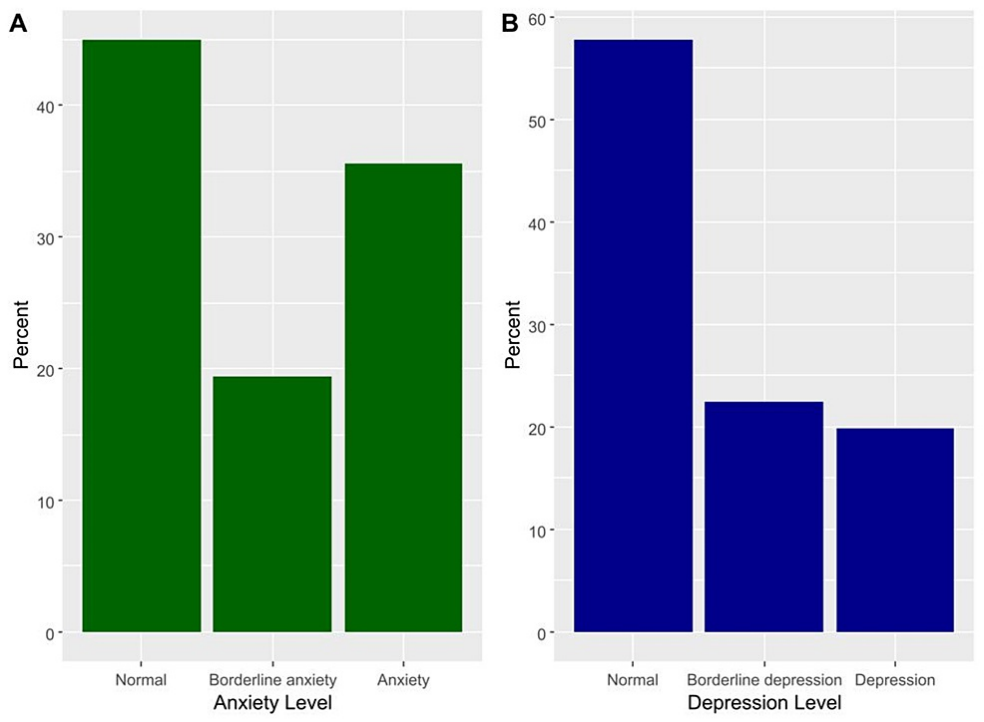
The percentages of different categories of anxiety (A) and depression (B) based on the HADS scale. HADS: Hospital Anxiety and Depression Scale

The median (IQR) NDI score was 12.0 (6.0, 22.0), and the domain showed excellent reliability (Cronbach’s alpha = 0.813). The median (IQR) scores of anxiety and depression were 8.0 (5.0, 12.0) and 7.0 (4.0, 10.0), respectively (Table 2). Severe to exaggerated neck disability was seen in 4.3% (Figure 1). Based on the HADS scale, borderline anxiety was prevalent among 19.4% and anxiety among 35.6% of participants (Figure 2A). Furthermore, 22.4% had borderline depression, and 19.8% of students had depression (Figure 2B).

**Table 2:** Descriptive and reliability analyses of the used scales. Cα: Cronbach’s alpha; NDI: neck disability index; IQR: interquartile range

Domain	Items	Cα	Median (IQR)	Range
NDI (Neck disability)	10	0.813	12.0 (6.0, 22.0)	0 to 86
HADS-A (Anxiety)	7	0.843	8.0 (5.0, 12.0)	0 to 21
HADS-D (Depression)	7	0.774	7.0 (4.0, 10.0)	0 to 19

Factors associated with neck pain

**Figure 3:**
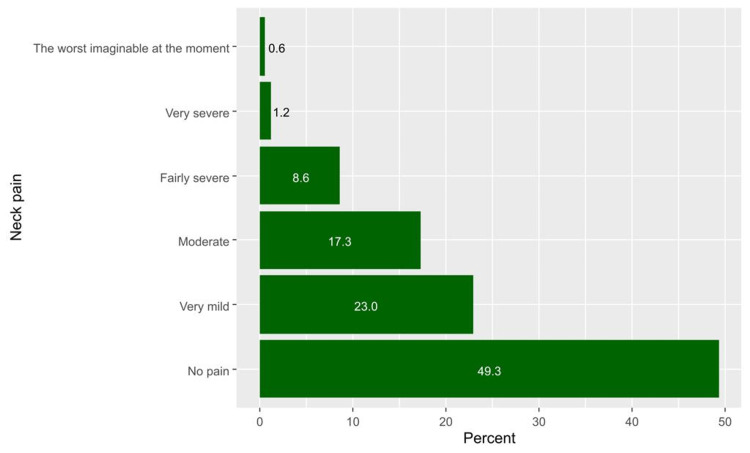
Participants' responses to their-self report perceptions regarding neck pain

**Figure 4:**
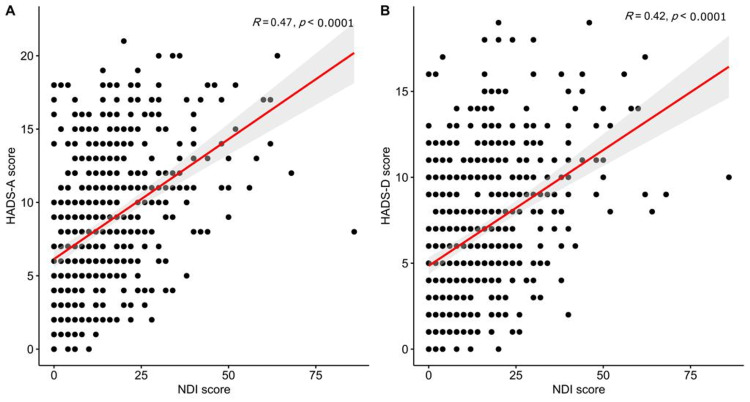
Bivariate correlations between the neck pain score, anxiety score (A), neck pain score, and depression score (B).

Generally, neck pain was prevalent among 258 participants, representing 50.7% of all the students (95%CI, 46.3 to 55.1). The categories of pain are demonstrated in detail in Figure 3. The median (IQR) neck pain scores were significantly higher among women (18.0, IQR 10.0-26.0 vs. 10.0, IQR 4.0-18.0 among men, p< 0.001), students who performed little or no exercise (14.0, IQR 6.0-24.0 for no exercise and 12.0, IQR 6.0-22.0 for little exercise vs. 10.0, IQR 4.0-18.0 for regular exercise, p = 0.016) and those who were consuming coffee more frequently (14.0, IQR 8.0-22.0 for >7 cups per week, 12.0, IQR 6.0-20.0 for 4 to 7 cups per weeks, and 14.0, IQR 6.0-25.5 for 1 to 3 cups per week vs. 8.0, IQR 4.0-19.0 for no coffee cups per week, p = 0.004, Table 3). Neck scores were also positively and significantly correlated with the scores of anxiety (R=0.47, p < 0.001, Figure 4A) and depression (R=0.42, p < 0.001, Figure 4B).

Factors associated with depression and anxiety

Results of the association analysis revealed that women had significantly higher median (IQR) scores of anxiety (10.0, IQR 6.0 to 14.0 vs. 7.0, IQR 4.0 to 11.0 among men, p< 0.001) and depression (7.0, IQR 4.0 to 10.0 vs. 6.0, IQR 3.0 to 10.0 among men, p< 0.001).

Anxiety scores were also significantly higher among students who performed no exercise (8.0, IQR 5.0 to 13.0) or occasional exercise (8.0, IQR 5.0 to 12.0) compared with those performing regular exercise (7.0, IQR 4.0 to 11.0, p = 0.020), and students with no exercise showed significantly higher depression scores (8.0, IQR 5.0 to 11.0) than those with occasional (6.0, IQR 4.0 to 10.0) or regular exercise (7.0, IQR 4.0 to 11.0, p = 0.002). Although anxiety scores differed significantly based on BMI categories (10.0, IQR 6.0 to 13.0 for underweight students, 8.0, IQR 5.0 to 12.0 for healthy students, 7.0, IQR 4.0 to 11.0 for overweight students and 8.0, IQR 4.8 to 12.2 for obese students), BMI levels did not imply differences in depression scores. No other socio-demographic characteristics were associated with anxiety or depression (Table 3).

**Table 3:** The scores of neck pain, anxiety (HADS-A), and depression (HADS-D) according to sociodemographic characteristics. HADS: Hospital Anxiety and Depression Scale

Parameter	Category	NDI score	Anxiety score	Depression score			
Median (IQR)	p-value		p-value		p-value		
Sex	Men	10.0 (4.0, 18.0)	<0.001	7.0 (4.0, 11.0)	<0.001	6.0 (3.0, 10.0)	0.005
	Women	18.0 (10.0, 26.0)		10.0 (6.0, 14.0)		7.0 (4.0, 10.0)	
Age	<20 years	14.0 (6.0, 30.0)	0.726	9.0 (6.0, 13.0)	0.118	8.0 (4.0, 9.0)	0.255
	20 to 23 years	12.0 (6.0, 22.0)		8.0 (5.0, 12.0)		6.0 (4.0, 10.0)	
	>23 years	12.0 (6.0, 20.0)		7.0 (4.0, 11.0)		6.0 (3.0, 9.0)	
BMI	Underweight	14.0 (6.0, 22.0)	0.65	10.0 (6.0, 13.0)	0.03	8.0 (5.0, 10.0)	0.169
	Healthy weight	14.0 (6.0, 22.0)		8.0 (5.0, 12.0)		6.0 (4.0, 9.0)	
	Overweight	12.0 (6.0, 22.0)		7.0 (4.0, 11.0)		6.0 (3.0, 10.0)	
	Obese	12.0 (4.0, 22.0)		8.0 (4.8, 12.2)		6.5 (4.0, 10.0)	
Marital status	Single	12.0 (6.0, 22.0)	0.061	8.0 (5.0, 12.0)	0.883	7.0 (4.0, 10.0)	0.383
	Married	18.0 (14.0, 32.0)		8.0 (6.0, 12.0)		7.0 (5.0, 13.0)	
Smoker	No	12.0 (6.0, 22.0)	0.979	8.0 (5.0, 12.0)	0.41	6.0 (4.0, 10.0)	0.975
	Yes	14.0 (6.0, 20.0)		8.0 (4.0, 11.0)		7.0 (3.0, 10.0)	
Do you exercise?	Yes, regularly	10.0 (4.0, 18.0)	0.016	7.0 (4.0, 11.0)	0.02	6.0 (3.0, 9.0)	0.002
	Yes, but occasionally	12.0 (6.0, 22.0)		8.0 (5.0, 12.0)		6.0 (4.0, 10.0)	
	No, not at all	14.0 (6.0, 24.0)		8.0 (5.0, 13.0)		8.0 (5.0, 11.0)	
Number of cups of coffee per week	None	8.0 (4.0, 19.0)	0.004	8.0 (4.0, 11.0)	0.148	6.0 (3.0, 9.0)	0.102
1 to 3	14.0 (6.0, 25.5)		9.0 (6.0, 12.0)		8.0 (5.0, 10.0)		
4 to 7	12.0 (6.0, 20.0)		8.0 (5.0, 12.0)		6.0 (4.0, 9.5)		
>7	14.0 (8.0, 22.0)		8.0 (5.0, 13.0)		7.0 (3.0, 10.0)		

In the multivariate analysis, we included the following independent variables: gender, exercise, BMI, and NDI score for anxiety risk factors for anxiety had the female sex (Beta = 1.50, 95% CI, 0.77 to 2.20, p < 0.001) and an increased neck pain score (Beta = 0.07, 95% CI, 0.04 to 0.10, p < 0.001). For depression, gender, BMI, and NDI score were used as independent variables. Results showed Higher neck pain scores were also independently associated with depression (Beta = 0.07, 95% CI, 0.04 to 0.09, p < 0.001, Table 4).

**Table 4:** Results of the multivariate regression analysis of the risk factors for anxiety and depression among students NS: the variable was not significantly associated with the outcome of the univariate analysis CI: confidence interval

Parameter	Category	Beta	95% CI	p-value
Anxiety				
Gender	Male	Ref	Ref	
	Female	1.50	0.77, 2.20	<0.001
Do you exercise?	Yes, regularly	Ref	Ref	
	Yes, but occasionally	0.39	-0.48, 1.3	0.380
	No, not at all	-0.26	-1.3, 0.75	0.609
BMI	Underweight	Ref	Ref	
	Healthy weight	-0.36	-1.3, 0.59	0.456
	Overweight	-0.86	-2.0, 0.26	0.132
	Obese	-0.11	-1.3, 1.0	0.856
NDI Score	Num	0.07	0.04, 0.10	<0.001
Depression				
Gender	Male	Ref	Ref	
	Female	-0.72	-1.3, 0.10	0.064
BMI	Underweight	Ref	Ref	
	Healthy weight	-0.61	-1.4, 0.23	0.154
	Overweight	-0.12	-1.1, 0.86	0.812
	Obese	-0.3	-1.3, 0.71	0.557
NDI Score	Num	0.07	0.04, 0.09	<0.001

## Discussion

Socio-demographic characteristics

Our study reveals that more than half of the participants were men, most of them aged between 20 to 23. Tantawy S et al. showed that the mean age of participants was 21 ± 1.9, and two-thirds were women [7]. Young S et al. also showed similar results with different sexes; however, the mean age was 49.8 [9]. These different results were attributed to diverse targeted populations. Regarding marital status, most participants were single similar to a study by Tantawy et al. [7]. This needs to be investigated more because there is not enough literature discussing it. Tantawy et al. and Dighriri Y et al. had a similar result to our study, where almost half of the participants had a normal BMI [7,2].

Moreover, in the Weleslassie G et al. study, the majority had a normal BMI [19]. Around a quarter of our study participants do not exercise, with similar results in the Dighriri Y et al. study [2]. In contrast, the Weleslassie G et al. study showed that approximately two-thirds of participants do not exercise [19]. BMI and exercise differences may depend on different lifestyles and cultural health thoughts. Smokers’ percentage did not exceed one-eighth of the total population size.

Descriptive and reliability analyses of the used scales

According to our participants, the most prevalent NDI disability scores were minimal and moderate than severe, respectively. Compared with a study by Young S et al. [9], the highest prevalence was moderate, minimal, then severe. In our study, severe to exaggerated disabling neck pain did not exceed 4.3% of the population. On the other hand, Young S et al. discovered that a quarter of the patients who visited his clinic had severe to completely disabling neck pain disability [9]. This may be explained by the sample difference in age and recruitment since the participants were older, had chief complaints of neck pain, and were recruited from the outpatient clinic [9]. In our study, anxiety and depression had approximately a prevalence of one-third and one-fifth, respectively. A study also reported a similar finding on patients with a history of neck pain. They demonstrated that the prevalence of anxiety and depression was identical to our study [11]. On the contrary, a survey of patients with chronic neck pain revealed an increase in the prevalence by one-third in anxiety and depression [10]. It is concerning that the bulk of psychological distress in our participants, who are otherwise considered healthy individuals, is increasing in most patients with neck pain.

Factors associated with neck pain

Our study indicates that the prevalence of neck pain is seen in one-half of all students, similar to the studies by Dighriri Y et al. and Weleslassie G et al. [2,19]. Neck pain is significantly higher among women. Du J et al. and Hendi O et al. reported this finding, demonstrating a higher neck pain among women [5,20]. This outcome contradicts Weleslassie G et al., who found that men are more affected [19]. There is a positive relation between neck pain and students who performed little or no exercise. This finding is consistent with that of Rose K et al. and Weleslassie G et al., which indicate the same relationship [3,19]. However, multiple studies by Dighriri Y et al. and Hendi O et al. revealed an insignificant relationship [2,20]. Exercise generally strengthens and stretches the neck muscle and provides natural support [19]. A significant positive correlation existed between those consuming more than seven cups of coffee weekly and neck pain. This finding does not support the previous results of Dighriri Y et al. and Algarni A et al., which indicate an insignificant relationship between the two [2,21]. However, our result agrees with Chang K et al., who suggested consuming caffeinated drinks as a potential contributing factor [22]. Therefore, it is likely that a connection exists between coffee consumption and neck pain, increasing the duration of studying. This could lead to developing bad habits such as inappropriate posture and immobility. Hindi O et al. showed no relation between anxiety and depression with neck pain [20]. This differs from our findings which revealed a significant relationship between anxiety and depression with neck pain. Similarly, Abdulghani H et al., Dighriri Y et al., Young S et al., and Al-Ghamdi S et al. found a significant relationship between psychological distress and physical pain [1,2,9,13]. A possible explanation is that anxiety and depression may be associated with psychosomatic manifestations such as neck pain.

Factors associated with depression and anxiety

The results reveal that women had a significantly higher rate of anxiety and depression. Abdulghani HM et al. and Al-Ghamdi S et al. also reported this finding and discovered that women had a higher rate of psychological distress [1,13]. This outcome is contrary to that of Blozik E et al., who showed a higher rate with men [11]. Hence, these results are likely to be related to the stress caused by the impact of the reproductive cycle [13]. Our study demonstrates that students who perform low physical activity have a significant risk of anxiety and depression. These results agree with those obtained by Paluska SA et al., which reveal that physical exercise can be a therapy for mild to moderate depression and anxiety [23]. This can be attributed to the release of endorphins and other chemicals in the brain enhanced by regular exercise.

Additionally, it distracts the mind from negative thoughts that aggravate anxiety and depression [24]. Zhao G et al. showed that BMI is significantly related to anxiety and depression [25]. This differs from the result presented in our research, which offers only a significant relationship between BMI and anxiety, with the highest score among underweight students. This might be accounted for by variations in the study population and potential confounding factors included in the study analyses.

Multivariate

The results of our study show that being a woman is an independent risk factor for anxiety. This finding is consistent with a study by Al-Ghamdi S et al. and Samuelsson et al. showing that women are at higher risk for psychological distress than men [13,26]. This can be attributable to the psychological predisposition of women to anxiety disorders and depression, which may be sex hormone-mediated. Furthermore, according to this study, neck pain with high scores is independently associated with anxiety and depression. Several reports have shown that neck pain is linked bidirectionally to anxiety and depression [8,27,28]. This observation may support the hypothesis that anxiety and depression may develop due to immunological changes produced by psychological stress and potential pain-related obstacles [29].

Limitations

One potential limitation of this study is the use of a self-reported questionnaire, which is influenced by the subject opinions of the student and may result in systemic bias. Another limitation is that the current study only assessed depression and anxiety as psychological variables. Other psychological variables may explain additional variance in the NDI and can be a topic for future research.

## Conclusions

The present research confirms that anxiety and depression significantly impact neck pain disability. In addition, being a woman is considered an independent risk factor for developing anxiety. Hence, it is essential to note that psychological distress can exacerbate neck pain and vice versa. Furthermore, both will affect academic performance and lead to decreased educational outcomes. We recommend conducting a cohort study on a targeted population with psychological distress and neck pain in the future to treat psychological distress and observe the presence or absence of improvements in neck pain and vice versa. Additionally, we encourage implementing our survey as a practical method to screen for psychological distress in students with neck pain.
